# Universal features of dendrites through centripetal branch ordering

**DOI:** 10.1371/journal.pcbi.1005615

**Published:** 2017-07-03

**Authors:** Alexandra Vormberg, Felix Effenberger, Julia Muellerleile, Hermann Cuntz

**Affiliations:** 1 Ernst Strüngmann Institute (ESI) for Neuroscience in Cooperation with Max Planck Society, Frankfurt/Main, Germany; 2 Frankfurt Institute for Advanced Studies (FIAS), Frankfurt/Main, Germany; 3 Institute of Clinical Neuroanatomy, Goethe University Frankfurt/Main, Germany; École Normale Supérieure, College de France, CNRS, FRANCE

## Abstract

Dendrites form predominantly binary trees that are exquisitely embedded in the networks of the brain. While neuronal computation is known to depend on the morphology of dendrites, their underlying topological blueprint remains unknown. Here, we used a centripetal branch ordering scheme originally developed to describe river networks—the Horton-Strahler order (SO)–to examine hierarchical relationships of branching statistics in reconstructed and model dendritic trees. We report on a number of universal topological relationships with SO that are true for all binary trees and distinguish those from SO-sorted metric measures that appear to be cell type-specific. The latter are therefore potential new candidates for categorising dendritic tree structures. Interestingly, we find a faithful correlation of branch diameters with centripetal branch orders, indicating a possible functional importance of SO for dendritic morphology and growth. Also, simulated local voltage responses to synaptic inputs are strongly correlated with SO. In summary, our study identifies important SO-dependent measures in dendritic morphology that are relevant for neural function while at the same time it describes other relationships that are universal for all dendrites.

## Author summary

Similarly to river beds, dendritic trees of nerve cells form elaborate networks that branch out to cover extensive areas. In the 1940s, ecologist Robert E. Horton developed an ordering system for branches in river networks that was refined in the 1950s by geoscientist Arthur N. Strahler, the Horton-Strahler order (SO). Branches at the tips start with order 1 and increase their order in a systematic way when encountering new branches on the way to the root. SO relationships have recently become popular for quantifying dendritic morphologies. Various branching statistics can be studied as a function of SO. Here we describe that topological measures such as the number of branches, the branch bifurcation ratio and the size of subtrees exhibit stereotypical relations with SO in dendritic trees independently of cell type, mirroring universal features of binary trees. Other functionally more relevant features such as mean branch lengths, local diameters and simulated voltage responses to synaptic inputs directly correlate with SO in a cell type-specific manner, indicating the importance of SO for understanding dendrite growth as well as neural computation.

## Introduction

Neurons of the central nervous system have a variety of shapes and possess dendritic trees that exhibit complex branching patterns. Apart from providing neurons with adequate connectivity, dendritic trees are not just simple passive signal conductors but are thought to be involved in sophisticated signal processing and neural computation [1,2]. Theoretical studies have suggested that dendritic morphology alone is able to influence a neuron's functional properties such as its firing patterns [3,4]. In particular, the topology of dendrites has been associated with strong effects on the temporal structure in the spiking behaviour [5,6]. Furthermore, the size of a neuron's dendritic tree, its diameter and its branching properties are all factors that influence the decay of synaptic signals on their way to the soma [7,8]. Understanding the principles governing dendrite morphology is therefore important for understanding neural computation. In order to better characterise and quantify dendritic branching structure, a number of branching statistics have been proposed [9,10]. Yet, these quantities exhibit strong correlations that are mostly unexplored [11]. Even when taken together, a commonly used set of existing branching statistics is not sufficient to cluster morphologies according to their given cell types [12].

In the following, we explore how sorting branching statistics by the precise order of the occurrence of nodes in a tree can increase the interpretative power of these statistical measures. Different methods have been developed to sort branches in dendrites. They mainly divide into those that start ordering the branches from the root, i.e. at the soma (centrifugal), and those that start from the terminal branches (centripetal) [13]. The centrifugal branch ordering method assigns a lowest order of 0 (or 1 depending on the definition) to the root, and increases the order by 1 at each branch point. Centrifugal branch ordering has found common use in many tree-like structures and has been specifically applied to dendritic trees on many occasions [14–20]. Among others, the maximum and mean branch order of dendritic trees have been used to measure dendritic tree complexity [21].

**Fig 1:**
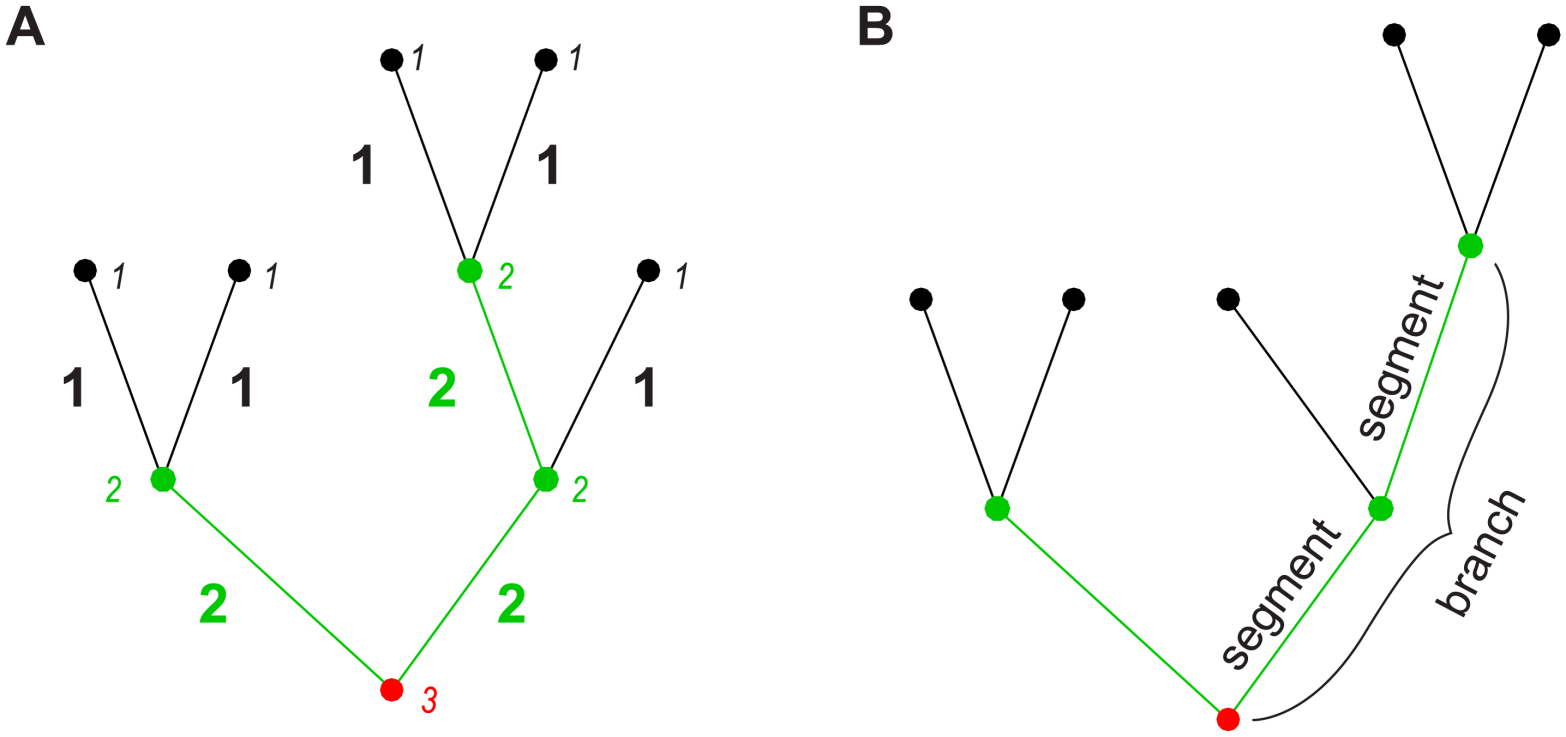
Depiction of the Strahler order (SO) scheme. **A**. Sketch to explain Strahler ordering (see main text). Small labels in italics denote node SO and large bold labels denote segment SO. Colours also indicate node and segment SO (black– 1, green– 2, red– 3). **B**. Sketch illustrating the difference between segments and branches regarding SO-sorted statistics.

Centripetal ordering schemes, on the other hand, have become increasingly common in recent years and we focus here on the so-called Horton-Strahler or Strahler order (SO), which was originally developed by Robert E. Horton as a stream ordering method for river networks [22]. This scheme was later refined and slightly modified to be objectively quantifiable by Arthur N. Strahler [23]. In Strahler's version, which we use in this study, all terminal branches are assigned order 1. The remaining orders are then constructed in an iterative way: When two branches of order *k* meet, the order of the parent branch is increased to order *k* + 1. When two branches of different order meet, the higher order prevails (see Fig 1A). The highest order of the tree always occurs at the root of the tree. The Strahler order of the root (or the maximal Strahler order assigned to a segment in the tree) is referred to as the Strahler number (SN) of the tree.

While SO started out in the field of geology as a stream classification scheme for river networks, it has since found use in many other scientific fields. For example, SO has been employed to quantitatively describe other tree-like structures such as bronchial trees or pulmonary arteries [24–27], as well as actual botanical trees [28–30]. Yet, it is important to note that certain findings that have been reported using Strahler orders have little descriptive power if the underlying structure is a binary tree. Most prominently this applies to the so-called “Horton’s law of stream numbers” [22], a power law between the counts of branches of a given SO and the SO itself. The slope of this distribution corresponds to the tree’s overall bifurcation ratio (i.e. the ratio of the number of branches between any two consecutive orders). This power law asymptotically approaches 4^1−*k*^ for SO *k* [31] and the corresponding bifurcation ratio converges to 4 [32,33]. The fact that its descriptive power is weak was only discovered much later in the 1990s using Monte Carlo methods [34]. These findings caused some discussions in the field and, interestingly, seem to be less known to the scientific community outside of hydrology. We will therefore comment on them in the following and distinguish results that are close to the expected case from ones carrying more descriptive power.

To our knowledge, Strahler’s method was first applied to the analysis of neuronal morphologies by Hollingworth and Berry, who used it to quantify and compare the branching structures of Purkinje and pyramidal cell dendrites in rats [35]. Later it was used to analyse the growth patterns of axons in cat visual cortex [36]. In recent years, SO also became popular as a measure of branching complexity in *Drosophila* dendritic arborisation (da) neurons of different classes [37–42]. Despite its wide applicability and relative simplicity, SO has not been studied in detail especially in relation to larger samples of neuronal cell types. Hence, this study uses Strahler's centripetal branch ordering method to investigate and describe the morphology of a variety of reconstructions of real dendrites as well as of synthetic model dendrites. We observe that other SO-sorted distributions of topological measures apart from Horton’s law are also nearly invariant to cell types and reflect universal features of binary trees. However, the metric measures that we studied exhibit differences between distinct cell types and may thus be used to quantify, categorise and better understand dendritic tree structure.

## Results

### Topological measures in a set of all possible binary trees of a given size and in random binary trees

**Fig 2:**
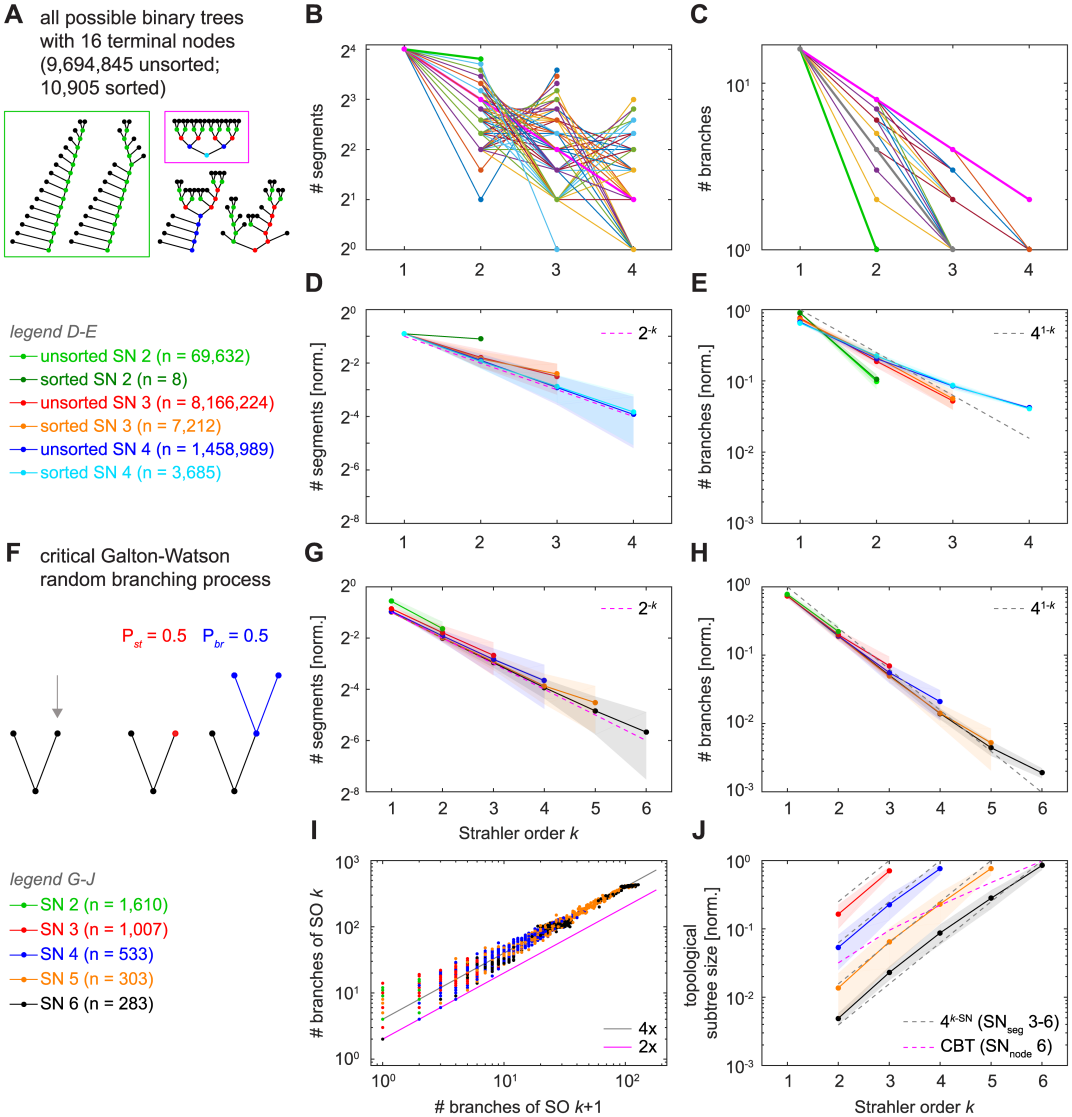
SO-sorted topological measures in binary trees. **A**. Sample binary trees taken from all possible 9,694,845 unsorted and 10,905 sorted trees of degree 16. Coloured dots denote node SO (black– 1, green– 2, red– 3, blue– 4, cyan– 5). **B**. Depiction of all possible distributions of segment numbers with SO in binary trees of degree 16; distributions for different trees are coloured differently. Bold green and bold magenta lines show distributions for the so-called “herringbone” tree (green box in A) and for the complete binary tree (magenta box in A), respectively. **C**. All possible distributions of branch numbers with SO in binary trees of degree 16. Bold green and bold magenta lines show distribution for the “herringbone” tree and for the complete binary tree, respectively. Bold grey line shows distribution for a tree that follows the trend of 4^1−*k*^ for SO *k* from dashed lines in E and H. **D**. Averages (lines with markers) and standard deviations (shaded areas) of normalised segment number distributions with SO for all possible sorted and unsorted binary trees of degree 16 divided into trees of segment Strahler number (SN) 2, 3, and 4. Dashed line indicates the distribution obtained from a complete binary tree (see main text). **E**. Similar to D but with normalised branch number distributions. Dashed line here indicates the asymptotic power relation obtained for large random binary trees (see main text). **F**. Sketch illustrating the Galton-Watson (GW) type random branching process. Terminal nodes either stop growing or branch out with probabilities *P*_*st*_ = 0.5 (red) and *P*_*br*_ = 0.5 (blue), respectively. **G, H**. Similar to D and E but for GW random binary trees divided into groups of SN 2–6. **I**. Branch bifurcation ratio between subsequent orders for the same GW random binary trees as in G and H. Magenta line indicates lower bound (see main text) and grey line denotes asymptotic bifurcation ratio for large random binary trees (see main text). **J**. Average (lines with markers) and standard deviations (shaded areas) of normalised topological subtree sizes for all topological nodes as a function of SO in GW random binary trees divided into SN 3–6. Magenta dashed line indicates relation obtained for a complete binary tree (CBT) of node SN 6 (see main text). Grey dashed lines represent mirror images (4^*k*−*SN*^, for SO *k* and SN 3–6) of the power relation in E (see main text). Note that in segment distributions per SO such as in D and G, nearly half of the segments are terminal segments (i.e. SO 1) in all cases, a property of binary trees.

Topological measures of dendritic trees can be calculated without any metric information, simply by analysing the succession of branch points (BPs) and termination points (TPs) in the tree. In the following, we first describe topological relationships with Strahler order (SO) while distinguishing between either segments (from BP—BP or BP—TP) or branches (consecutive segments) of a given SO *k* (Fig 1B). In order to better interpret results from SO-sorted relationships in dendritic trees, we first obtained results from the set of all possible binary trees with a given number of nodes (Fig 2A–2E). After calculating all unsorted binary trees with 16 terminal nodes, we also calculated the reduced set of all sorted binary trees in which the ordering of subtrees at each node is discarded by allowing permutations at all BPs to identify topologically equivalent trees (see Methods; the two trees in the green box in Fig 2A are topologically equivalent since they can be transferred into one another by rotations of subtrees). For such small trees (enumerating this set quickly becomes computationally expensive for larger trees as it grows exponentially with tree size) that are typical for some real dendrites (e.g. dentate gyrus granule cells), the distributions of SO-sorted segments (Fig 2B) and branches (Fig 2C) could vary widely in different binary trees. At one extreme, the so-called “herringbone” tree that is maximally asymmetric (Fig 2A, sample trees in the green box) showed a flat line with SO-sorted segment number (Fig 2B, green bold line) with SO values restricted to 1 and 2. At the other extreme, the complete binary tree (CBT), which is completely filled with nodes on every level (Fig 2A, sample tree in the magenta box), doubled the number of nodes at every step that decreased SO, leading to a 2^−*k*^ trend (Fig 2B, magenta bold line). By contrast, the mean values for these distributions (Fig 2D and 2E) followed much clearer trends. With increasing SN (the maximal SO assigned to a segment in the tree), the average number of segments per SO for all possible trees of degree 16 was well described by the same 2^−*k*^ trend predicted by the complete binary tree (Fig 2D, magenta dashed line). This is partly explained by the fact that we grouped trees according to their SN: Maximally asymmetric herringbone trees are characterised by an SN of 2. By contrast, symmetric CBTs exhibit maximal SN values for their respective number of nodes with distributions of 2^−*k*^. Asymmetric trees naturally have lower SN values, therefore groups of trees with larger SN will be more symmetric and behave more like the complete binary tree.

The possible distributions for the number of branches per SO also varied widely (Fig 2C) while the averages (Fig 2E) tended to a specific trend, in this case 4^1−*k*^ (Fig 2E, grey dashed line). This is a consequence of Horton’s law and the 4^1−*k*^ power described in the introduction.

Generally, their topology has previously linked dendritic trees to certain classes of random binary trees [43]. We therefore compared our results from the set of all possible binary trees of degree 16 with a set of random trees produced by a critical Galton-Watson (GW) random branching process [44] that generates a distribution of trees of different sizes (Fig 2F). Such a GW process has been used in the past to generate a number of topologically distinct binary trees and to model dendrite branching patterns [45]. We obtained a large number of GW binary trees by randomly choosing to either terminate or branch further at every new terminal node in an iterative manner. The branching process was terminated when no further branching occurred or when a total number of 800 nodes was reached. The number of segments distribution grouped by SN (Fig 2G) again followed the 2^−*k*^ trend that would be expected for the CBT and was very similar to that seen in Fig 2D (see above). As expected from Kirchner [34], the number of branches per SO (Fig 2H) tightly approximated the same relationship as observed for all possible binary trees of degree 16 (Fig 2E). Due to the larger magnitude of GW random binary trees compared to the set of all possible binary trees of degree 16, the trends were much more pronounced. The 4^1−*k*^ trend in SO-sorted branch numbers can further be observed in the branch bifurcation ratios (Fig 2I), a related topological measure that compares the number of branches of SO *k* with that of the next higher order *k* + 1. Since bifurcations do not allow ratios lower than 2, this lower limit value is true for the CBT where SO increases at every single branch point (Fig 2I, magenta line; see also Fig 2C where the line is an upper bound). In this representation, most data points are evenly scattered around the average bifurcation ratio of 4 (coefficient of determination *R*^2^ = 0.9925), the asymptotic bifurcation ratio calculated previously for a large number of nodes [32–34] (see Discussion). Finally, we studied the topological subtree sizes capturing the number of daughter BPs and TPs for all BPs of a given SO in our GW random binary trees (Fig 2J). Since all nodes with SO 1 are TPs, the values here start at SO 2. We found an exponential increase in subtree size with SO that was not well described by the topological subtree sizes expected for a CBT (Fig 2J, magenta dashed line, root node SN 6) but was rather well approximated by 4^*k*−*SN*^ (Fig 2J, grey dashed line shown for all segment SN values), a mirror image of the relation that we found for the number of branches.

### Topological measures and optimal wiring

**Fig 3:**
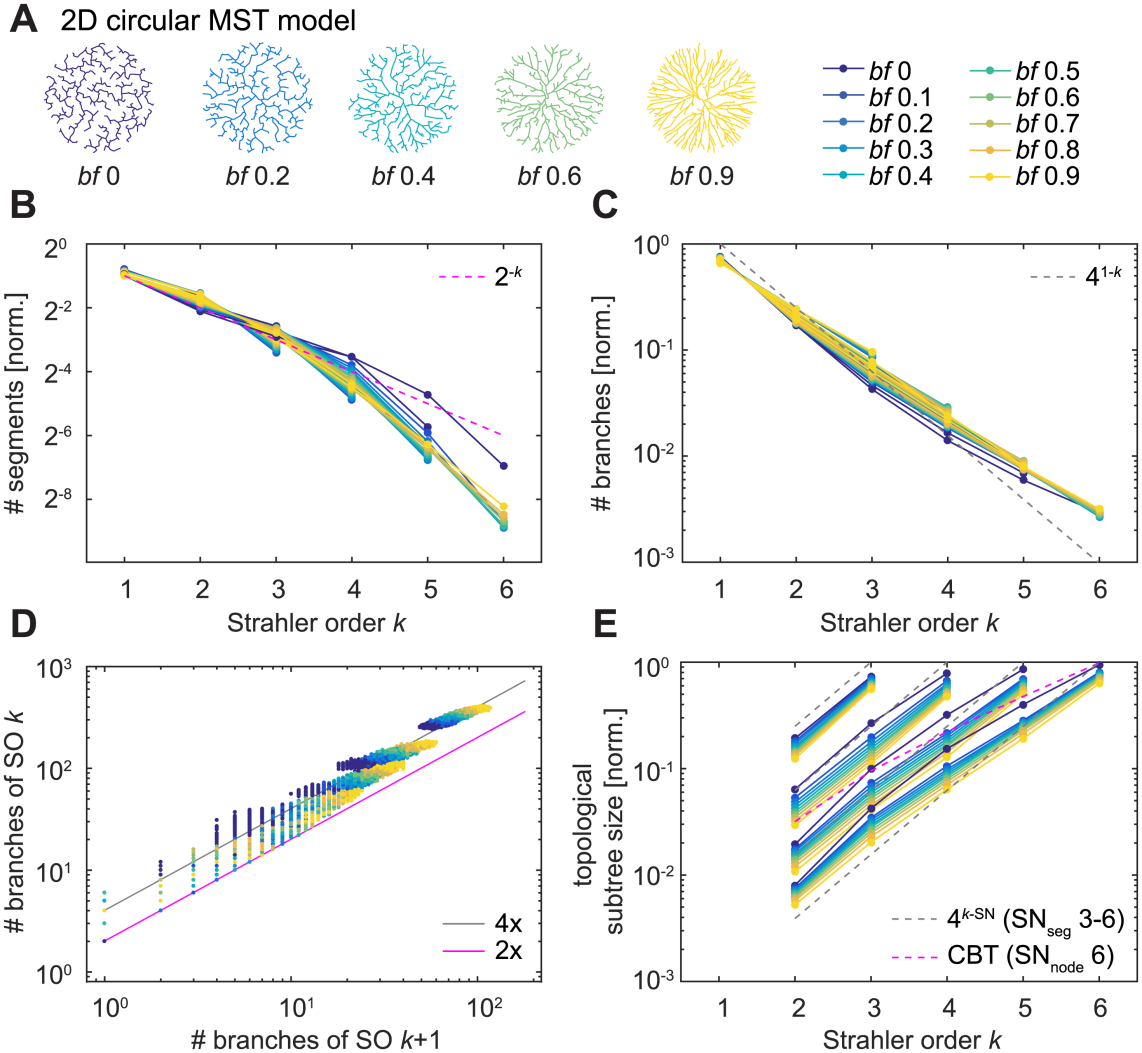
SO-sorted topological measures in minimum spanning trees (MSTs). **A**. Example synthetic MST model dendrites (2D circular morphology) generated with 500 target points and differing balancing factors (*bf*). Colours and legend to the right indicate *bf* in the remaining panels. **B—E**. Similar plots to Fig 2G–2J. For B, C, and E, each line represents the mean of 614–970 (SN 3), 398–940 (SN 4), 625–967 (SN 5), and 248–964 (SN 6) model trees per *bf*. For better clarity, standard deviations are not shown.

Since dendrites serve the particular purpose of network connectivity, we investigated how their resulting branching pattern distributions compare to those of random binary trees. We have previously introduced a minimum spanning tree (MST) based growth algorithm that connects a set of target points to generate synthetic dendrites. Meeting the requirements for dendritic connectivity, such MST-based model trees guarantee short total dendritic lengths and with one parameter, the balancing factor (*bf*), also increasingly prohibit long paths along the tree towards the root to reduce conduction times in the neural circuit [46]. When the resulting synthetic dendrites are restricted to binary trees by ruling out more than two daughter branches at a branch point, MST-based model trees represent a specialised, optimally-wired distribution over the set of all possible binary trees: Since they are grown on the basis of random target points distributed within a specific area, they rely on metric information to produce the resulting topology. For this reason, the binary trees resulting from the MST process might be distributed in a highly selective manner. In order to test this, we examined two-dimensional circular morphologies that varied the two parameters *bf* and the number of target points (in order to obtain trees of SN 3–6, see Methods for details; Fig 3A). The results for such MST-based model trees were similar to the set of all possible binary trees of degree 16 and the GW random binary trees (Fig 3B–3E), indicating that the specific sample of binary trees that perform optimal wiring is not easily distinguishable from a set of random trees by the SO-based metrics we used here. The branch bifurcation ratio (Fig 3D) in MST-based model trees was also tightly scattered around a ratio of 4 (*R*^2^ = 0.9814, over all trees) in agreement with the results from the random binary Galton-Watson model trees. Overall, *bf* 0 –the pure minimum spanning tree—seemed to be a special case potentially because it represents the least symmetric tree.

### Topological measures in real dendrites

**Fig 4:**
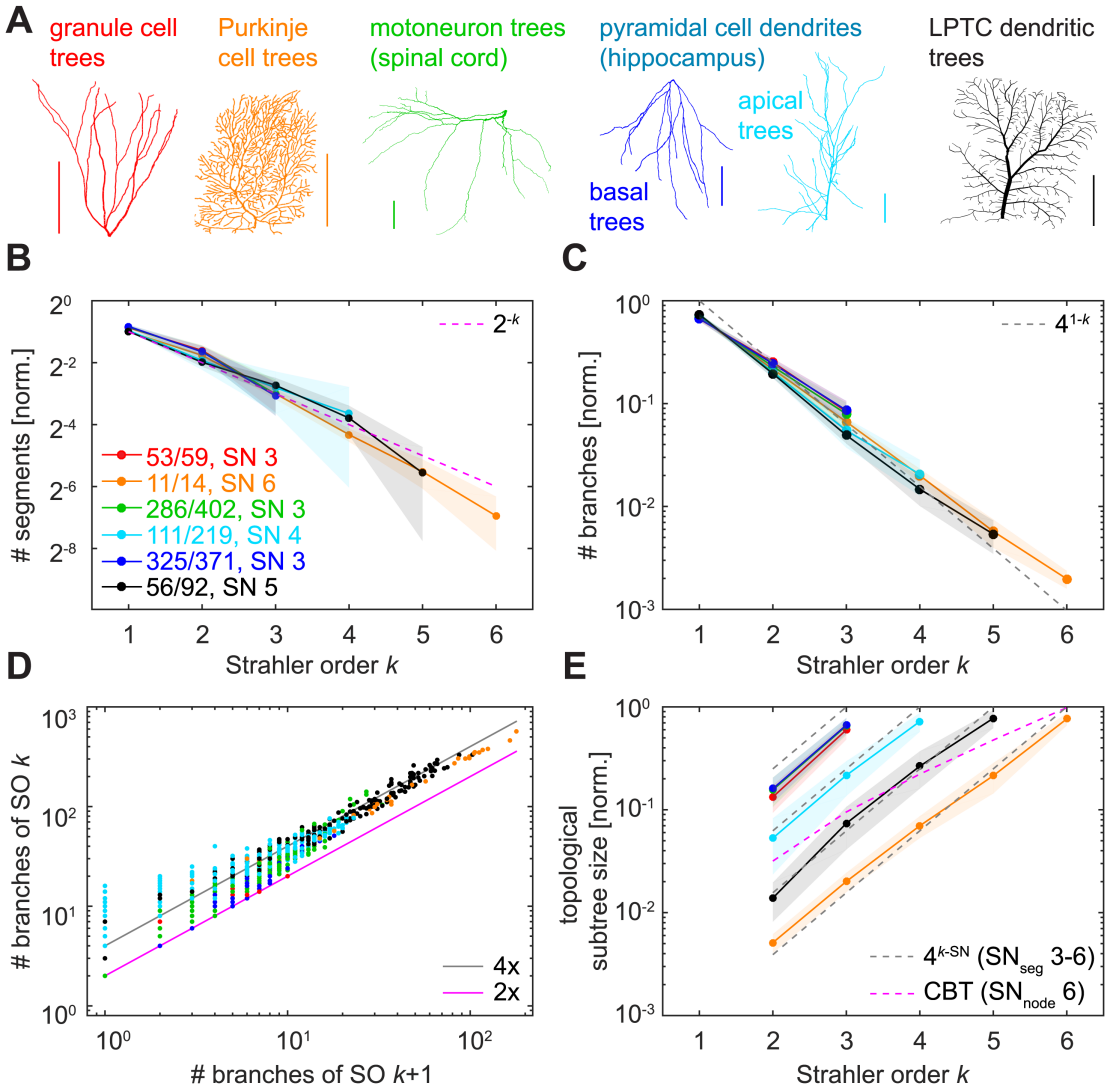
SO-sorted topological measures in real dendritic trees. **A**. Example reconstructed morphologies of the six dendrite types that were analysed in this study. Colours indicate cell types in the remaining panels (red—granule cell; orange—cerebellar Purkinje cell; green—spinal cord motoneuron; cyan—hippocampal pyramidal cell, apical dendrite; blue—hippocampal pyramidal cell, basal dendrite; black—lobula plate tangential cell, LPTC). Scale bars always 100 μm. **B—E**. Similar plots to Fig 2G–2J. Legend in B shows number of dendritic trees used for the plot compared to the total number of trees analysed for that dendrite type. For each cell type, only averages of trees with one given (i.e. the most abundant) SN were plotted, except for panel D in which all investigated dendritic trees of a cell type were used. Shaded areas denote standard deviations.

We then investigated the same topological measures in six real reconstructed dendrite types (Fig 4A) and found that they, too, behaved very much like the binary tree models shown above (Fig 4B–4E). Firstly, the normalised number of segments per SO approximated 2^−*k*^ for SO *k* (Fig 4B) with a slope between -0.89 and -1.20 for a fit by linear regression in binary logarithmic space in the six groups of reconstructed dendrites (*R*^2^ > 0.9686 for slope fits in all groups). Secondly, the expected invariance of the SO-sorted number of branches matching a 4^1−*k*^ decay for SO *k* (Fig 4C) was pronounced and we found linear slopes ranging from -0.45 to -0.54 in the log10 scale (*R*^2^ > 0.996 for slope fits in all groups). Thirdly, the bifurcation ratios for reconstructions of real dendrites (Fig 4D) exhibited linear regression slopes varying between 2.23 for dentate gyrus granule cells and 3.77 for lobula plate tangential cells (LPTCs; Table 1), and the fit for all lumped data was 3.44. However, the coefficient of determination for a fit of 4 was *R*^2^ = 0.9387. Finally, the subtree sizes distributions (Fig 4E) followed the 4^*k*−*SN*^ increase with SO *k* observed previously, with linear slopes on the logarithmic average data ranging from 0.54 to 0.65 (*R*^2^ > 0.9898 for slope fits in all cases).

**Table 1:** Linear regression fits for overall bifurcation ratios of reconstructed neuronal morphologies (see main text).

Granule cells	Purkinje cells	Motoneurons	Pyramidal cells, apical	Pyramidal cells, basal	LPTCs
2.23	3.12	3.65	3.38	2.68	3.77

Taken together, these analyses show that the SO-sorted topological measures yielded very similar distributions over a wide range of possible binary tree samples including all real dendritic trees, rendering these measures unsuitable for morphological classification, cf. [34].

### Metric measures in real dendrites

**Fig 5:**
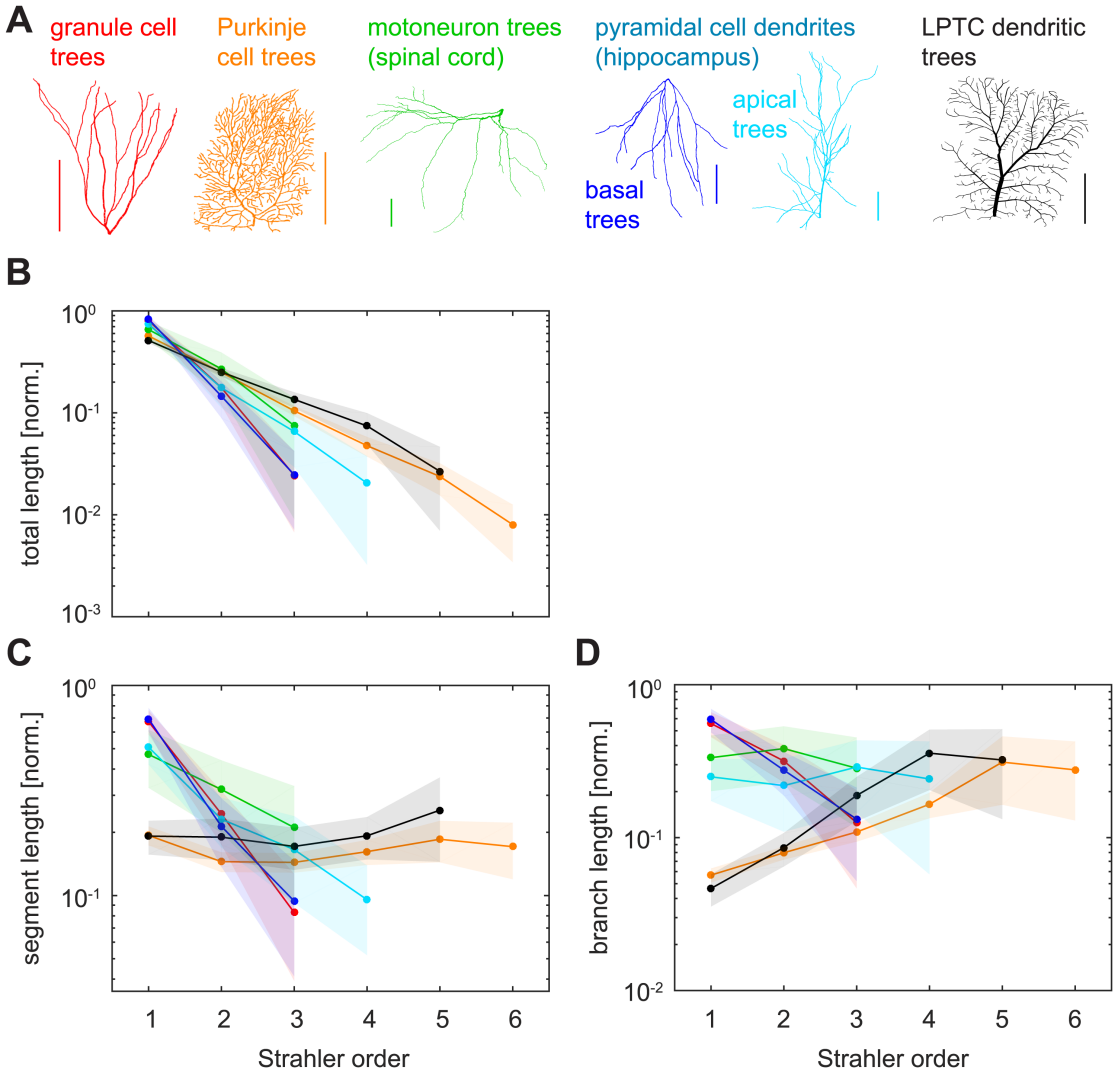
SO-sorted metric measures in real dendritic trees. **A**. Legend; same as in Fig 4A. **B**. Average normalised total dendritic length as it distributes per SO in the reconstructed cell types from Fig 4. **C**. Average normalised segment lengths as they differ for different SO values. **D**. Average normalised branch lengths as they differ for different SO values. Shaded areas denote standard deviations.

Dendritic trees in the brain are embedded in 3D tissue, adding metric information such as *X*, *Y* and *Z* coordinates as well as diameter values to the nodes in their tree structures that are not captured by the topologies of abstract binary trees. While any local branching statistics could exhibit interesting relationships with SO, we focused on the distributions of total dendritic length, mean segment lengths, mean branch lengths, and branch diameters, as well as basic passive electrotonic properties. The amount of total dendritic length as a function of SO followed an approximately exponential decay for all reconstructed dendrite types that we analysed, as seen by straight lines in the semi-logarithmic plots (Fig 5B). However, the slope of the decay was clearly different for dendrites of various types: The decay was slower for planar, 2D morphologies (e.g. LPTCs, Purkinje cells), for which 50–60% of the total dendritic length was SO 1 (terminal segment or branch length). By contrast, the decay was faster in 3D morphologies such as dentate gyrus granule cell dendrites and basal pyramidal dendrites with the combined terminal branch length making up more than 80% of the total wiring length. Linear regression fits on the semi-logarithmic data yielded slope values between -0.31 and -0.76 (*R*^2^ > 0.9892 in all cases).

Normalised mean segment length distributions with SO also varied pronouncedly with cell type (Fig 5C). Mean segment lengths in planar lobula plate tangential cell (LPTC) dendrites and Purkinje cells were nearly constant over all SOs. By contrast, the distributions for 3D cells featured longer mean segment lengths in the terminals, followed by a rapid decrease over the remaining SOs in dentate granule cells (linear regression fit slope -0.45, *R*^2^ = 0.9996), basal pyramidal dendrites (-0.43, *R*^2^ = 0.9894), apical pyramidal trees (-0.23, *R*^2^ = 0.9748), and motoneurons (-0.17, *R*^2^ = 0.9995). SO-sorted distributions of mean branch lengths varied even more strongly between different dendrite types (Fig 5D). Some distributions increased exponentially with SO up until a certain point; e.g. Purkinje cells and LPTCs (exponential increase until their peak in the second-to-last order with linear regression slopes in logarithmic space of 0.18 and 0.30, respectively; *R*^2^ = 0.9766 and *R*^2^ = 0.9978). Others decreased exponentially (granule cells -0.32, *R*^2^ = 0.9805, basal pyramidal dendrites -0.33, *R*^2^ = 0.9999), and yet others still were nearly constant (motoneuron dendrites and apical pyramidal dendrites).

### Metric measures in the MST model

**Fig 6:**
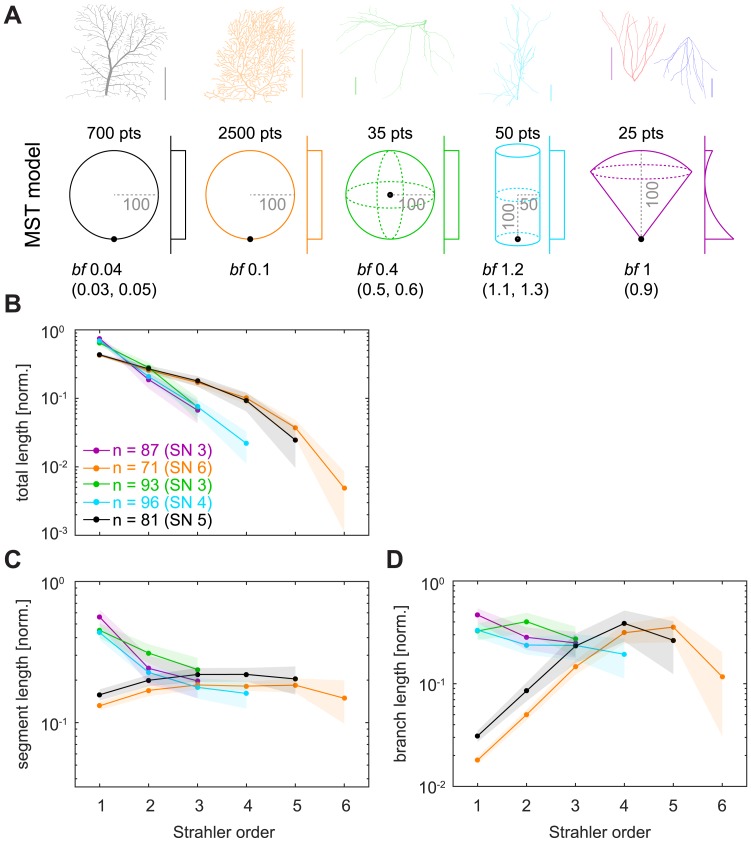
Simulations of SO-sorted metric measures using synthetic trees. **A**. Top row shows the same dendritic tree reconstructions as in Fig 4A. Bottom row shows the respective geometric arrangements for which MST models reproduce the SO-sorted statistics observed in real cells (Fig 5). Number of target points (*pts*), balancing factor (*bf*) including its possible ranges in parentheses, and indications of sizes (grey with dashed lines) are given as numbers. Black dots show root node location. 2D or 3D spanning areas are sketched in the respective colours and distributions of target points are sketched next to the spanning areas (uniform for all but the rightmost model; see Table 2 for details). Granule cell and basal pyramidal dendrites were represented by the same model (violet). Colours indicate the respective models in the remaining panels (see Table 2 for details). **B—D**. Similar plots as in Fig 5B–5D. Shaded areas denote standard deviations.

In order to understand which features of the dendritic geometry led to the differences that we observed in SO-sorted metric measures, we designed simple MST-based model trees that reproduced the properties observed in Fig 5 (Fig 6). The MST results depended strongly on the spatial distribution of the target points that were to be optimally connected. We matched the results for SO-sorted measures seen in real dendrites by modulating basic parameters of the MST model such as hull shape, root node location, balancing factor *bf*, number of target points *pts* and the mode of target point distribution (Fig 6A, bottom row; Table 2). The slope of exponential decay observed previously for SO-sorted total dendritic length (Fig 5B) was replicated when using 2D vs 3D spanning hulls for the MST-generated trees (Fig 6B). However, the details of the distributions were not entirely captured in the 2D models (LPTC and Purkinje cell models), indicating that potentially more complex features of morphology determine the traits observed in Fig 5B. Both the nearly constant mean segment length distribution seen in LPTCs and Purkinje cells (Fig 6C) and the more complex relationships of branch length distributions there as well as in motoneurons and apical dendrites of pyramidal cells (Fig 6D) could be replicated easily by geometric adjustments to the MST models. However, granule cell and basal pyramidal dendrites with their similar distributions in Fig 5 required precise reconstruction also of the inhomogeneous quadratic target point density along the height of the cone in which the MST-based model trees were grown (Fig 6A, violet model). Even then, the model did not capture the exponential decrease of segment and branch length seen in Fig 5C and 5D very well. These findings suggest that complex cell type-specific differences in the geometry of the growth process determine the different SO-sorted distributions of metric branching statistics that we observed in Fig 5.

**Table 2:** Parameters for synthetic MST-based model trees in Fig 6.

	LPTC model (SN 5)	Purkinje cell model (SN 6)	Motoneuron model (SN 3)	Apical pyramidal dendrite model (SN 4)	Granule cell / basal pyramidal dendrite model (SN 3)
**Hull shape**	2D circle (radius 100)	2D circle (radius 100)	3D sphere (radius 100)	3D cylinder (radius 50, height 200)	3D cone (height 100)
**Root location**	Offset to edge [0 -100 0]	Offset to edge [0 -100 0]	Centre [0 0 0]	Centre of base [0 0 -100]	Cone vertex [0 0 0]
***bf* used (other possible *bf*)**	0.04 (0.03–0.05)	0.1	0.4 (0.4–0.6)	1.2 (1.1–1.3)	1 (0.9–1.0)
**Target points distribution**	Uniform	Uniform	Uniform	Uniform	Inhomogeneous along cone height; with probability values corresponding to ${\left(\frac{height-65}{100}\right)}^{2}$
**Number of target points**	700	2500	35	50	25

### Functional compartmentalisation of SO-sorted tree segments

**Fig 7:**
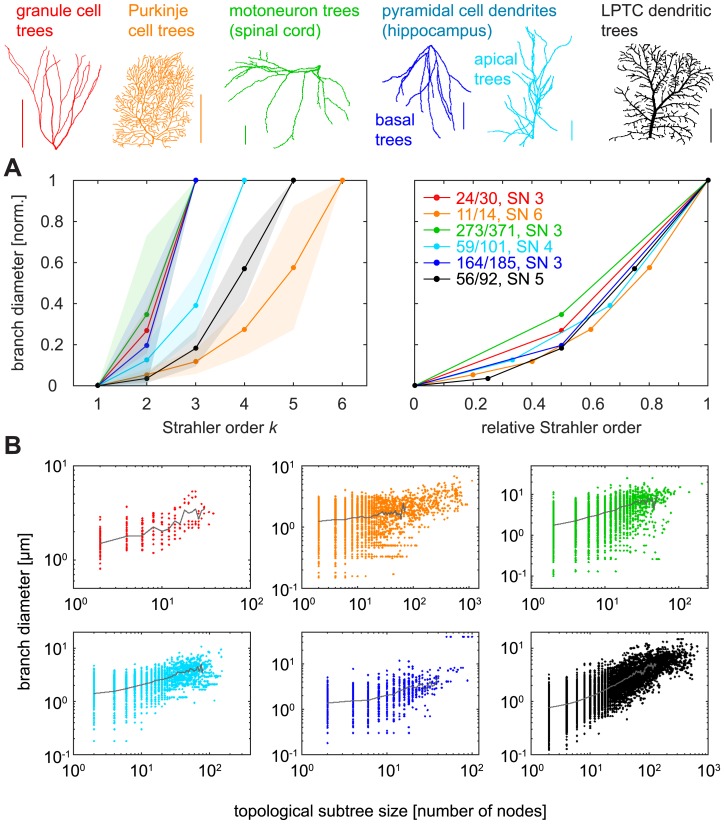
Branch diameters in reconstructed dendritic trees as a function of SO. **A**. *Left*: Average normalised diameters (between minimum and maximum values) for nodes of different SO in reconstructed dendritic trees and their standard deviations (shaded areas); *right*: same, but with SO values normalised between 0 and 1 (standard deviations omitted for clarity). **B**. Diameter values as a function of topological subtree size for all branch points in the trees, divided in panels for each cell type. Grey line corresponds to the average diameter for branch points of the same subtree size and is shown until the number of data points available for mean calculation was less than 0.1% of the data points for topological subtree size 2. Different colours denote data from different cell types (see legend at top of figure).

It is well known that terminal segments of dendritic trees exhibit the smallest diameters [8,47,48] and that this contributes to the local input resistance being highest there [47,49]. At the same time, even short branches in close proximity to the soma typically reach the smallest diameters, suggesting a centripetal increase in diameter rather than a regular taper away from the root. In the following, we investigate a potential relationship between SO and these features that are expected to be important for neuronal computation. The relationship between diameters and SO was striking and followed a similar trend in all cell types (Fig 7A). This increase of diameters with SO would be consistent with quadratic fits (*R*^*2*^ > 0.9956 for all morphologies), which is in agreement with our predictions that quadratic tapers optimise current transfer in dendritic trees [8,47]. Since we showed that SO correlated with subtree sizes (Fig 4E) it was not surprising that diameters also weakly correlated with subtree sizes (Fig 7B).

**Fig 8:**
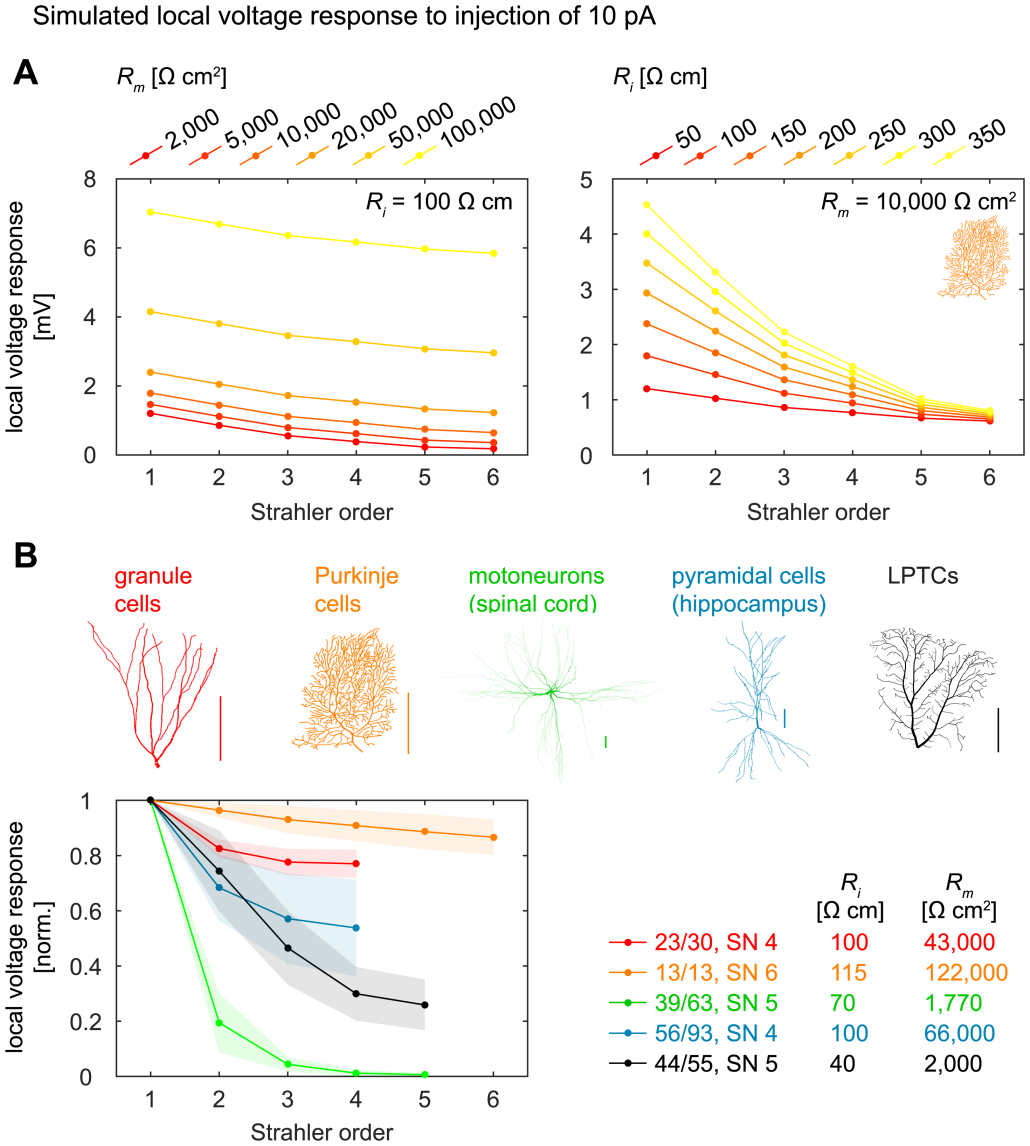
Simulated local synaptic voltage responses as a function of SO. **A**. Average simulated local voltage responses to small steady-state synaptic currents (10 pA) injected into topological nodes of SO *k* in entire Purkinje cell dendrites (n = 13) of SN 6 with different passive properties. *Left*: Variation of specific membrane resistance *R*_*m*_ with fixed specific internal resistivity *R*_*i*_; *right*: variation of *R*_*i*_ with fixed *R*_*m*_. The Purkinje cell dendrites corresponded to the whole dendrite of the cell, with no additional change of morphology (see Methods). Standard deviations not shown for better clarity. **B**. Similar to A, but for the different cell types. *R*_*i*_ and *R*_*m*_ values were taken from the literature (see Methods). Top shows legend: entire-dendrite morphologies were taken, as in A (see Methods). Shaded areas denote standard deviations.

As mentioned above, local diameter values are influential in the processing of synaptic inputs and small diameters result in large local input resistances with strong voltage deflections for a given synaptic input. Additionally, electrotonic properties depend in part on dendritic topology [5,6,50]. We therefore also analysed simulated voltage responses to small steady-state synaptic currents (10 pA) in passive entire dendrites (see Methods) and indeed found strong relationships with SO (Fig 8A for Purkinje cells with different passive properties and Fig 8B for the different cell types and their respective passive properties). Interestingly, all cell types apart from Purkinje cells exhibited strong relationships with SO. This matches well with our previous finding that diameters in Purkinje cells were not optimised to transfer currents to the root [8] and could indicate a particular functional role of the Purkinje cell dendrite that is to date not understood.

## Discussion

In this study, we showed that the branching structures of real dendritic trees are indistinguishable from random binary trees when using four different Strahler order (SO) sorted topological measures. The universal relationships that we uncovered were only partly known for random binary trees and they are important for understanding general features of topological measures when used to characterise dendritic trees. By contrast, we showed that SO-sorted metric measures differ according to cell types and therefore may be useful to categorise or quantify dendritic tree structures and their respective functions.

### SO-sorted topological measures reflect universal properties of binary trees

Regardless of the method used to select samples out of the set of all binary trees, whether it was the average of all sorted or unsorted binary trees, randomly selected binary trees, or trees that guaranteed optimal wiring (MST-based model trees), we found that the relationships between topological measures and SO followed universal trends. This was also the case for all dendritic trees we studied, independently of cell type, and spanned measures from branch numbers distributions and bifurcation ratios to distributions of segment numbers and subtree sizes.

As mentioned above, it has previously been shown analytically that the mean number of branches of a given Strahler order *k* in random binary trees asymptotically tends to 4^1−*k*^ as the number of terminals goes to infinity [31], with a corresponding overall bifurcation ratio of 4 [32,33]. Additionally, most binary trees lie close to a bifurcation ratio of 4 [34], and it has been suggested that it is inherent to the definition of Strahler order that no binary tree can depart indefinitely far from this branch number power law [51]. In fact, Van Pelt et al. [52] have shown that there are only a few unique values that the overall branch bifurcation ratio (i.e., the averaged ratio between the number of branches of any two successive SOs) can take for the set of all possible binary trees of a given size (e.g. 7 unique values for the 98 sorted possible binary trees of degree 10, or 18 unique values for the 10,905 sorted trees of degree 16). It is therefore not surprising that we found this relation to be true in Galton-Watson (GW) random trees (Fig 2H, dashed grey line) and in the set of all possible binary trees of a given size (Fig 2E).

The universality of this distribution of branch numbers and bifurcation ratios extends to a wide range of tree-like structures in nature that most likely do not reach an exact bifurcation ratio of 4 because of their relatively small size. In our study, overall bifurcation ratios varied from 2.23–3.77 for different dendritic tree types. In river networks, they commonly range from 3–5 [22,32]. Similar bifurcation values as well as the general exponential decay relation with increasing SO have also been observed with bifurcation ratios between 3.05–3.61 in the dog bronchial system [24], 2.99–3.10 in the human pulmonary arteries [25], 3.41–4.12 in networks of conducting particles in a dielectric liquid [53], and 3–5.76 in social networks [54]. They are also present in several different species of botanical trees, with bifurcation ratios ranging from 3–5.18 [28–30]. As we demonstrated here, this general range is similar for neuronal dendritic trees. Previously, the number of branches per SO in Purkinje cell dendrites has been found to decay exponentially with a linear regression slope for log-transformed data of -0.52 for cells of SN 6 and -0.46 for cells of SN 7 [35], which are values in a range similar to what we find in the reconstructed dendrites investigated in this study. Furthermore, Binzegger et al. [36] investigated axonal branching in cat visual cortex and found it to be topologically self-similar, i.e. with a bifurcation ratio that is similar between each two consecutive orders and amounts to 3.32 for both spiny and smooth axons (*r* = 0.99).

While a significant number of publications have explored branch number distributions and bifurcation ratios in detail, the universal trend of the SO-sorted subtree size distribution is more elusive. Subtree sizes at branch points of a given SO increased exponentially with SO in a similar manner for all groups of binary trees studied here, including those of real dendrites. Subtree sizes are connected to distributions of number of branches, since the number of TPs and BPs in a tree can never be lower than the number of branches. In fact, subtree sizes appeared to mirror the distributions of number of branches with a steady 4^*k*−*SN*^ for SO *k*. We assume that those relations could be proven analytically using similar methods as in [31–33].

Surprisingly, a distribution very similar to that of SO-sorted topological subtree size (Fig 4E) was found when calculating the ratio of total dendritic length contained in the subtree of topological nodes of a given SO (S1 Fig). Such an exponential increase of local subtree weight with SO may have functional implications for electrotonic compartmentalisation and synaptic current transfer, in particular since we also found a relationship between subtree sizes and local diameters (Fig 7B).

Furthermore, the distribution of segment numbers with SO was very similar for all binary trees we studied. It has previously been found to decay in an exponential manner in Purkinje cells [35,55], and we observed that this relation holds true for many additional dendrite types. Here, on average, binary trees behaved similarly to the complete binary tree (CBT), characterised by its doubling of segments at every level where SO decreases. This is explained by the fact that any tree of a given *SN* > 2 must necessarily contain a certain number of complete subtrees in order to increase SO at branch points until the corresponding SN is reached (see Results).

The apparent universality and low variability of the SO-sorted segment and branch numbers and the branch bifurcation ratio in reconstructed dendrites can thus be explained by the fact that these statistics are universal for most binary trees and therefore of low descriptive power, cf. [34]. But it is important to note that if dendrites had followed a particular given blueprint, the distributions could have been widely different (Fig 2B and 2C). In this way, however, the universal property of SO-sorted topology in binary trees renders those distributions essentially useless for quantifying dendritic trees in a meaningful way (see also “Concluding remarks on Strahler order and neuronal dendrites” below).

### SO-sorted metric measures are cell type-specific

The results were very different for metric measures, which exhibited strong correlations with SO that were cell type-specific in many cases. For example, we found that SO-sorted distributions of total dendritic length followed a pronounced exponential decay that seemingly depended on the dimensionality of the dendrites, since planar dendritic trees (e.g. Purkinje cell dendrites) revealed more shallow slopes than dendrites that extended into 3D space (e.g. granule cells). In line with this, SO-sorted total length in frog retinal ganglion cell (RGC) axons has been reported to decrease in a mostly exponential fashion, with 40–50% of the total length contributed by terminal segments and with a slope that closely resembles our LPTC data (black line in Fig 5B) [56]. We were able to reproduce this dimensionality effect using simple MST-based model trees based on optimal wiring principles for the different dendrites, where the slope of decay was additionally related to the *bf* parameter (see Fig 6B). The total dendritic length distribution does not denote a universal relation as seen with the number of segments or branches but is reflective of specific morphological attributes such as how planar a dendrite is. Similar observations were made for SO-sorted mean segment and branch lengths. Interestingly, we were unable to accurately replicate the exponential decay of mean segment and branch lengths with SO in basal pyramidal and granule cell dendrites using simple MST models. This might indicate that these trends are a result of the particular features of these cells, and the measures that we studied here could indeed help to better classify dendrites of such cell types.

While dendritic trees followed Horton's law of stream numbers as expected, not all of them obeyed Horton's law of stream lengths, which expresses the mean branch length of SO *k* as a direct geometric series starting with the mean branch length at SO 1 [22]. In dendrites, we found highly varying SO-sorted distributions of mean branch lengths, as opposed to the exponential increase postulated by Horton's law. For real dendritic trees that are flat and were represented with a low balancing factor in our respective MST models (e.g. LPTCs and Purkinje cells), mean branch length did appear to follow Horton’s law of stream lengths to some extent: The distribution increased exponentially with SO until it peaked in the second-to-last order, i.e. close to the root. Similarly, Hollingworth and Berry [35] observed an exponential increase in the mean lengths of branches of successive SO in rat Purkinje dendrites, and an increase was also reported by Yen et al. in frog RGC axons [56]. For granule cell dendrites and basal pyramidal dendrites, however, the distribution exhibited an exponential decrease. Since river networks are planar, it is conceivable that Horton's law of stream lengths applies only to 2D trees. Meanwhile, motoneurons and apical pyramidal dendrites appeared to fall somewhere in between those extremes and did not exhibit clear trends.

The most striking correlation was found between local branch diameters and SO. While the general presence of a correlation may be reasonably expected in a centripetal ordering system since diameter has been well established to taper towards the terminals of neurons, there seems to be no precedent of an investigation of SO-sorted branch diameter in neurons, apart from a report that diameter increases with SO in frog RGC axons [56]. Because diameter values strongly determine electrotonic properties of neurons, we also studied the consequences for synaptic integration and found that simulated local voltage responses to synaptic currents correlated strongly with SO. Interestingly, similar correlations between diameters and SO have been observed in other tree structures in nature. Branch diameters appear to increase exponentially with SO in the bronchial tree of dogs [24] and in botanical trees [29,30]. Additionally, topological subtree size was strongly correlated with SO and to a lesser extent with diameter, showing a functional relevance of SO.

Potentially, the variations in SO-sorted statistics could also reflect differences in the growth process: While some neurons such as granule cells could “blossom” like flowers, elongating mainly the branches close to the soma, others such as Purkinje cells could mostly develop by growing new terminal branches and densifying the fixed space they occupy. In line with this, Van Pelt et al. [43] demonstrated that the geometry of guinea pig Purkinje cells can be replicated using a dendritic growth model that favours branching at distal locations.

### Concluding remarks on Strahler order and neuronal dendrites

SO has advantages as well as disadvantages when compared to other branch ordering systems. Uylings et al. [13] reviewed different ordering methods for dendrite branching and concluded that the Strahler method should preferentially be used when studying branching patterns that are very asymmetric, or when studying tree structures with a very extensive branching pattern such as Purkinje cells, because the tree's Strahler number SN is not as high as the maximum branch order in the centrifugal system. A caveat of the method is its sensitivity especially to the addition or loss of terminal segments, as these can alter the order of many branches in the tree. It has furthermore been suggested that centripetal ordering schemes are superior when examining branch probabilities in distal regions of the dendrite, but that centrifugal ordering of branches is useful for branching structures near the soma or root of the tree [16].

The main usage of Strahler ordering applied to neuronal morphology in the literature so far is concerned with the distribution of the SO-sorted number of branches. It has been used for classification of branching complexity of Drosophila da neurons, as mentioned in the Introduction (e.g. [37,38,40–42]), and various other studies used that distribution to ascertain differences in “branching complexity” in various cell types, mostly aforementioned da neurons or Purkinje cells, between different conditions such as wild-type vs. knock-out cells (e.g. [38,40,57–60]). In all of these studies, the authors used the actual number of branches, which would have varied compared to the normalised number. However, since the normalised branch numbers distribution is on average very similar in different groups of trees (dendritic or otherwise), it is quite redundant to compare the number of branches of different orders between cells because the slope of the curve is not likely to differ very much, as discussed above. In fact, in most of those studies, it would have been enough to compare just the number of terminal segments without using any branch ordering system (or, perhaps, with the additional information of the SN): Where actual numbers were given in various publications [38,57–59,61,62], we plotted the number of branches per SO and saw that they did indeed follow the characteristic exponential decay that was close to a 4^1-*k*^ slope for SO *k* if values were normalised to the total number of branches. In most of the publications surveyed, significant differences in branch numbers appeared only in SO 1, i.e. the number of terminals. We argue that using SO only in this way is limiting and redundant, and that more interesting results might emerge if Strahler ordering were used more often in conjunction with metric measures such as total length per SO (only seen in one publication pertaining to axonal morphology; [56]). Some studies, e.g. [60], did investigate total length between two groups of cells, but there may be benefits to looking at the SO-sorted total length distribution as well as it might offer information regarding the dendritic locations where the length changes most significantly. Further metric SO-sorted measures such as mean branch and segment length might also be of use to quantify differences in morphology and branching complexity.

Our study demonstrates that one has to take great care when interpreting the topology of trees using SO because of the many universal features that make such quantification potentially useless. This stresses the importance of using computational models to better interpret the results from quantitative measures that are commonly used in neuroscience. Finally, we observe that SO can be a useful tool to classify tree structures and their functional relevance when used in conjunction with adequate metric information.

## Methods

All analyses were performed using the TREES toolbox, an open-source software package for MATLAB (Mathworks, Natick, MA, USA), which provides tools for analysing and generating neuronal morphologies [46,63] (www.treestoolbox.org). We used functions of the TREES toolbox as well as additional custom MATLAB code to generate model dendritic trees, to pre-process reconstructed real dendrites, and to determine and visualise the various Strahler order-sorted branching statistics. A function *strahler_tree* to calculate Strahler order (SO) values for each node in a tree was implemented and will be made available in the TREES toolbox package.

### A set of all possible binary trees

In order to obtain the set of all possible binary trees of a given maximal size, we calculated all possible instances using a formal language defined in the following way: We started with the string ‘BTT’ representing a binary tree on 3 nodes, where ‘B’ stands for branch point and is followed by two subtrees and ‘T’ is a termination point. Recursively, all ‘T’ elements were then replaced by new branches ‘BTT’, one by one resulting in as many next generation trees with each two additional nodes. After calculating all trees of a given generation, all exact duplicates were removed. This procedure was then continued until the target tree size was obtained. Notice that the number of binary trees of a given size grows exponentially with the number of nodes (yielding 1, 2, 5, 14, 42, 132, 429, 1,430, etc. as the number of trees in each generation). For example there exist 9,694,845 binary trees on 31 nodes (16 ‘T’s), which was at the limit of the computing power available to us. We chose the set of all possible binary trees of degree 16 because we wanted to investigate a set that would include a complete binary tree. See Fig 2A for resulting sample binary trees. Note though that many of these trees were equivalent in terms of the topological structure since permutations of subtrees at every branch point are actually equivalent (for example a tree ‘BBTTT’ is equivalent to ‘BTBTT’; see also green box in Fig 2A for two topologically equivalent binary trees). Apart from the unsorted set of trees described above (i.e., the set which allows multiple topologically equivalent binary trees with differing ‘B’-and-‘T’ strings), we therefore calculated the reduced (sorted) subset that contained only topologically unique trees. To determine this sorted subset, the *sort_tree* function from the TREES toolbox was used to arrange all trees of a newly generated generation such that heavy subtrees would always be represented first in the ‘B’-and-‘T’ strings, thus enabling us to exclude duplicates. A few remaining duplicates were identified by an exhaustive search comparing all permutations of all subtrees at each branch point in the remaining trees. In this way, we obtained all 10,905 [52,64] sorted binary trees on 31 nodes (16 ‘T’s). Finally, tree structures were generated from the resulting ‘B’-and-‘T’ strings using the TREES toolbox function *BCT_tree*. The resulting trees were then analysed further using the TREES toolbox.

### Galton-Watson (GW) random branching model

We generated random binary trees by using a critical Galton-Watson random branching process [44]. This growth process starts with one terminal node at generation 1. For each successive generation, each terminal node of the previous generation is treated in one of the following two ways: (1) with probability *P*_*st*_, growth stops there; (2) with probability *P*_*br*_, the node becomes a branch point, and edges are added with two new terminal nodes (Fig 2F). Terminal growth was previously found to be compatible with the observed topologies in real dendrites [65]. A Galton-Watson process is called critical if *P*_*st*_ = 0.5 and *P*_*br*_ = 0.5. We used custom MATLAB code to generate 10,000 random topological trees, terminating each growth process in the cases when the tree size reached 800 nodes.

### Synthetic minimum spanning tree (MST) based model neurons

Spatially embedded minimum spanning trees (MST) take into account optimal wiring considerations that are important for real neurons. MST models (here we use the *MST_tree* function from the TREES toolbox) were previously used successfully to model a large palette of dendrites [7,46,47,66–68] and axons [69]. These models generate tree structures that connect a set of target nodes to minimise the total dendrite length as well as the cost for short paths from the target nodes to the root along the tree. Target nodes are given to the algorithm and may be chosen to be distributed inside specified two- or three-dimensional areas according to certain rules (e.g. uniform distribution when coordinates for target nodes are generated as uniformly distributed random numbers on a specified interval), and will end up being the branch points, termination points, and continuation points of the tree. The cost for short paths from target nodes to the root is weighted with the balancing factor (*bf*), with typical values between 0 and 0.9 in real dendrites. When *bf* is low, the total wiring cost is kept to a minimum and the resulting tree approaches a minimum spanning tree. When *bf* is high, direct paths from target points to the root are given more importance than the pure conservation of wiring.

To study topological measures in MST models (Fig 3), we uniformly distributed a number of random target points (30, 150, 500 or 1,200, in order to obtain as many trees as possible for SN 3, 4, 5, and 6, respectively) in a two-dimensional circular area of 10,000 μm^2^ with a root node located in the centre. 1,000 MST-based model trees were generated for each combination of *bf* (between 0 and 0.9, in steps of 0.1) and target point number. Here, the *MST_tree* function was constrained to bifurcations (option *‘-b’*), enforcing the generation of binary trees. The resulting trees were repaired using the *repair_tree* function, which assigns a number to every node in the tree according to a predefined standard, and all nodes from the root to the first branch point were deleted, forcing every tree to start with a branch point to best match the concept of binary trees. If nodes were deleted in the process, the *repair_tree* function was applied once again to update node indices accordingly.

In order to investigate the source of the cell type-specific differences in SO-sorted metric measures of real dendrites (Fig 5), we modelled the SO-sorted distributions by finding the simplest MST model parameters necessary to approximate those distributions (Fig 6). The initial model conditions were similar to the model described above with simple two- or three-dimensional (depending on the real cells) round spanning fields and a root node located in its centre. The complexity of the hull, the root location and the statistics of the target point distributions were adjusted manually in a trial-and-error procedure in an attempt to best reproduce—with as few modifications as possible—all SO-sorted branching statistics as seen in the real dendrites. In all cases, the balancing factor and number of target points were varied to best match the real dendrite statistics and SN values. Specific parameters for each of the five models are described in Table 2 and Fig 6A (bottom row). Binary synthetic trees were obtained in a similar manner as described above. 100 synthetic trees were generated for each of the models.

### Reconstructions of real morphologies

Reconstructions of real dendritic morphologies (examples see Fig 4A) were taken from the *NeuroMorpho*.*Org* database (accessed on 26/11/2015) with the exception of the blowfly LPTCs [66], which were taken directly from the set of sample cells in the TREES toolbox (see Table 3 for details). A preselection of neurons was conducted according to metadata filtering on the *NeuroMorpho*.*Org* website (Table 4). In brief, we restricted the dataset to neurons with complete dendrites that were part of the control conditions in any given experiment. All preselected neurons were manually inspected in three-dimensional view. Since the metadata is self-reported by the contributing labs, the quality of “complete” reconstructions may differ from dataset to dataset. Many reconstructions exhibited sudden step-like jumps in depth, a common problem in three-dimensional reconstructions. We excluded such dendrite reconstructions with insufficient quality by visual inspection but no objective criteria to appropriately quantify the quality of the tracings were used. Reconstructions of typically three-dimensional neurons (e.g. granule cells, pyramidal cells) that did not or did only slightly extend into the third dimension were also excluded from the analysis. The number of suitable cells left for each cell type can be found in Table 3. Despite the fact that our *NeuroMorpho*.*Org* metasearch required diameter information, we found that some of the remaining cells had poorly reconstructed diameter values (i.e., nearly constant diameter for all nodes). These cells were still used for most analyses in this study but were excluded from the SO-sorted branch diameter computation, which was therefore performed on a lesser number of dendritic trees per cell type (see Table 3).

**Table 3:** Overview of reconstructed morphologies.

Cell type (brain area)	Number	Number, with diameter	Trees	Trees, with diameter	Taken from archive (species)
Granule cells (hippocampus)	60	30	59	30	Cho (mouse), Lee (mouse), Vuksic (mouse)
Motoneurons (spinal cord)	70	66	402	371	Alvarez (rat), Ascoli (mouse), Burke (cat), Cameron (cat), Chmykhova (turtle), Fyffe (cat), Rose (cat)
Purkinje cells (cerebellum)	13	13	14	14	Dendritica (rat), Dusart (mouse), Martone (mouse, rat)
Pyramidal cells (hippocampus)	195	93	219 (apical), 371 (basal)	101 (apical), 185 (basal)	Amaral (rat), Ascoli (rat), Barrionuevo (rat), Beguin (mouse), Gulyas (rat), Jaffe (rat), Johnston (rat), Kim (mouse), Korte (mouse), Spruston (rat), Turner (rat), Wittner (guinea pig), Wu (mouse)
LPTCs (lobula plate)	55	55	92	92	[66] (blowfly)

**Number**–number of suitable reconstructed cells.

**Number, with diameter**–number of reconstructions used for diameter computations.

**Trees**–number of suitable reconstructed dendritic trees with *SN* > 2 after soma and axon deletion.

**Trees, with diameter**–number of reconstructed dendritic trees used for diameter computations.

**Taken from archive (species)**–*NeuroMorpho*.*Org* archive name and species.

**Table 4:** Selection criteria in *NeuroMorpho*.*Org* database metadata search.

anatomy → cell type → principal cell →	granule cell	Purkinje cell	motoneuron	pyramidal cell
anatomy → brain region →	hippocampus	cerebellum	spinal cord	hippocampus
completeness → structural domain →	{dendrites, soma, axon OR dendrites, soma, no axon}			
completeness → physical integrity → search by dendrites →	complete			
completeness → morphological attributes →	diameter, 3D, angles			
experiment → experimental condition →	control			

We then deleted all cell regions that were not labelled “dendrite” (i.e., axon and soma regions). Deletion of a single cell's soma resulted in multiple dendritic trees (Table 3) if there were several primary dendrites emerging from the soma. We pooled data for all dendritic trees of a cell type for analysis, with the exception of pyramidal cells, where we analysed apical and basal trees as separate groups due to their very different morphologies. All dendritic trees were then repaired with the TREES toolbox function *repair_tree*, replacing branch points with more than two daughters with multiple consecutive bifurcations to restrict ourselves to binary trees (no multifurcations allowed). Furthermore, we identified the first branch point following the root (if it was not the root node already) and deleted all previous nodes. A deletion was followed by another application of the *repair_tree* function. This deletion step was performed to ensure that all analysed dendritic trees were binary trees that started with a branch point. However, it must be noted that this is an alteration of the original morphology and leads to exclusion of data concerning the length and presence of small branches of higher Strahler orders (e.g. dendrites that emerge from the soma as a single branch before they first bifurcate).

### Strahler order (SO) analysis

Our morphometric analysis distinguishes between dendrite segments and branches. A segment is defined as a piece of dendrite connecting two consecutive topological nodes in the tree (BP—BP or BP—TP). Consecutive segments (moving from terminals towards the root) of the same SO form one branch of that order. A branch may consist of only one segment (e.g., all terminal segments are also terminal branches), but often consists of multiple segments, especially for higher orders (Fig 1B, the tree contains three SO 2 segments, but only two SO 2 branches, one of which is composed of two segments). We additionally distinguish between node and segment SO to determine the highest order in a tree, its Strahler number (SN). We call the highest SO assigned to a segment in a tree its segment Strahler number. The highest SO of any node in a tree is its node Strahler number. In most cases, these two are interchangeable. However, when two segments of the same order meet at the root node of the tree, they are different (e.g. in Fig 1A: segment SN = 2, but node SN = 3). For node-based measures (subtree size, branch diameter, local voltage response), we forced functions to consider the root node’s SO as equal to the tree’s segment SN in the few cases where node and segment SN differed. This step did not significantly alter the resulting SO-sorted measures but was performed to ensure that all trees sharing a common segment SN would also display a common number of data points (from SO 1 to the order corresponding to the segment SN) that were averaged for visualisation in the figures.

The **topological measures** we analysed are the number of segments, number of branches, branch bifurcation ratio, and the size of topological subtrees. These measures do not require any metric information and therefore they enable us to compare these distributions across not only reconstructed neurons and synthetic MST morphologies, but also Galton-Watson random branching model trees as well as the set of all possible binary trees of degree 16. Except for Fig 2B and 2C, SO-sorted distributions of number of segments and branches were normalised (to the total number of segments and branches in the tree, respectively) to be able to compare distributions of trees of different sizes. The branch bifurcation ratio was obtained by visualising the number of branches of any SO *k* with the number of branches at SO *k* + 1. When bifurcation ratios between all consecutive orders in a tree are similar, the tree is said to be topologically self-similar. Hence, the bifurcation ratio provides information about the fractal degree of a structure. We calculated overall bifurcation ratios by performing a linear regression on the number of branches of SO *k* against the number of branches of SO *k* +1 for all trees of a dendrite type. Topological subtree sizes counting the number of BPs and TPs for any BP in the tree should intuitively increase for centripetal branch ordering schemes such as SO. Topological subtree sizes were also normalised (to the total number of topological nodes in the tree) and used for Figs 2, 3 and 4.

The following **metric measures** were determined as a function of SO for reconstructions of real neurons and for their respective MST models: total dendritic length, mean branch length, and mean segment length. Also, branch diameters were investigated in the reconstructed morphologies where diameter values were available. Normalised total lengths per SO express the proportion of total dendritic length in each SO. Normalised mean branch and segment lengths per SO were obtained by dividing the total length value for a given SO by the total number of branches or segments respectively for that given SO value. These values were further divided by the sum of the average branch or segment length values of all SO (integral under the curve is then 1 in all cases) to keep the resulting distributions in the same range. Mean branch diameters per SO were calculated by taking the sum of the diameters of all nodes with a given SO and dividing that by the number of nodes of that given SO. Normalised relative values between 0 and 1 were obtained by setting the lowest mean diameter value for any order (this was always SO 1) to 0 and the highest mean diameter value (this was always the order corresponding to the tree’s SN) to 1.

**Electrotonic measures** in passive dendritic trees were calculated on a slightly differently pre-processed dataset because we took values for *R*_*i*_ and *R*_*m*_ from the literature that described whole cells instead of dendritic trees that were once part of a bigger dendritic field. We therefore took all neurons with reasonable diameter information (see Table 3, “Number, with diameter”), removed soma and axon regions in such a way that the dendrite would stay connected and not result in multiple dendritic trees, and repaired the dendrite with the *repair_tree* function, which may result in a small change of morphology and topology.

To simulate the average local voltage response to an injection of 10 pA into a topological node of SO *k*, the dendritic tree’s electrotonic signature given its specific membrane conductance *G*_*m*_ = 1/*R*_*m*_ and specific internal resistivity *R*_*i*_ was determined using the *sse_tree* function. *R*_*m*_ and *R*_*i*_ for the different cell types were taken from the literature [70–73] for Fig 8B. The diagonal of the resulting matrix contained the local input resistances in MΩ of every node in the tree. These values were averaged between all topological nodes that shared the same SO. In order to simulate the local voltage response to a small steady-state synaptic current injection of 10 pA, the values were divided by 100 and the unit became mV. For normalisation, all values were divided by the average value for the terminal nodes (SO 1). Three motoneurons (‘Alvarez-Control-Cell-1.CNG’, ‘Alvarez-Control-Cell-2.CNG’, and ‘Alvarez-Control-Cell-3.CNG’) had to be excluded from this simulation because of their high number of nodes.

### A note concerning data visualisations

All figures of SO-sorted distributions show averages of trees that share the same segment SN. In this way, patterns in the data are not skewed by averaging over model trees or real dendrites in a sample that have different SN values. For reconstructed morphologies, we chose the most abundant SN value for each cell type for visualisation, hence only the subset of the data where all trees share that SN value was plotted (e.g. see Fig 4B: for granule cells (in red), there were 59 dendritic trees analysed, but the graph shows only the average of those 53 trees in the subset of all granule cell dendritic trees that had segment SN 3).

## Supporting information

S1 Fig Length of subtree per SO for topological nodes in real dendritic trees. Average (lines with markers) and standard deviations (shaded areas) of normalised subtree lengths for all topological nodes as a function of SO in real dendritic trees. Colours indicate cell types (for legend see Fig 4A). Values were normalised by expressing them as a ratio of total dendritic length. Grey dashed lines same as in Fig 4E.
(TIF)

## References

[1] Euler T, Denk W. Dendritic processing. Curr Opin Neurobiol. 2001;11: 415–422. 11502386 10.1016/s0959-4388(00)00228-2

[2] London M, Häusser M. Dendritic computation. Annu Rev Neurosci. 2005;28: 503–532. doi: 10.1146/annurev.neuro.28.061604.135703 16033324 10.1146/annurev.neuro.28.061604.135703

[3] Mainen ZF, Sejnowski TJ. Influence of dendritic structure on firing pattern in model neocortical neurons. Nature. 1996;382: 363–366. doi: 10.1038/382363a0 8684467 10.1038/382363a0

[4] Connors BW, Regehr WG. Neuronal firing: Does function follow form? Curr Biol. 1996;6: 1560–1562. doi: 10.1016/S0960-9822(02)70771-9 8994812 10.1016/s0960-9822(02)70771-9

[5] Van Elburg RAJ, Van Ooyen A. Impact of dendritic size and dendritic topology on burst firing in pyramidal cells. PLoS Comput Biol. 2010;6: e1000781 doi: 10.1371/journal.pcbi.1000781 20485556 10.1371/journal.pcbi.1000781 PMC2869305

[6] Van Ooyen A, Duijnhouwer J, Remme MWH, Van Pelt J. The effect of dendritic topology on firing patterns in model neurons. Netw Comput Neural Syst. 2002;13: 311–325. 10.1088/0954-898x/13/3/304 12222816

[7] Platschek S, Cuntz H, Vuksic M, Deller T, Jedlicka P. A general homeostatic principle following lesion induced dendritic remodeling. Acta Neuropathol Commun. 2016;4: 19 doi: 10.1186/s40478-016-0285-8 26916562 10.1186/s40478-016-0285-8 PMC4766619

[8] Bird AD, Cuntz H. Optimal current transfer in dendrites. PLoS Comput Biol. 2016;12: e1004897 doi: 10.1371/journal.pcbi.1004897 27145441 10.1371/journal.pcbi.1004897 PMC4856390

[9] Ascoli GA, Alonso-Nanclares L, Anderson SA, Barrionuevo G, Benavides-Piccione R, Burkhalter A, et al Petilla terminology: nomenclature of features of GABAergic interneurons of the cerebral cortex. Nat Rev Neurosci. 2008;9: 557–568. doi: 10.1038/nrn2402 18568015 10.1038/nrn2402 PMC2868386

[10] Torben-Nielsen B, Cuntz H. Introduction to dendritic morphology Cuntz H, Remme MWH, Torben-Nielsen B, editors. The Computing Dendrite. New York, NY: Springer New York; 2014 doi: 10.1007/978-1-4614-8094-5

[11] Costa LDF, Zawadzki K, Miazaki M, Viana MP, Taraskin SN. Unveiling the neuromorphological space. Front Comput Neurosci. 2010;4: 150 doi: 10.3389/fncom.2010.00150 21160547 10.3389/fncom.2010.00150 PMC3001740

[12] Polavaram S, Gillette TA, Parekh R, Ascoli GA. Statistical analysis and data mining of digital reconstructions of dendritic morphologies. Front Neuroanat. 2014;8: 138 doi: 10.3389/fnana.2014.00138 25538569 10.3389/fnana.2014.00138 PMC4255610

[13] Uylings HBM, Smit GJ, Veltman WAM. Ordering methods in quantitative analysis of branching structures of dendritic trees. Adv Neurol. 1975;12: 247–254. 1155264

[14] Coleman PD, Riesen AH. Environmental effects on cortical dendritic fields. J Anat. 1968;102: 363–374. 5656134 PMC1231476

[15] Lindsay RD, Scheibel AB. Quantitative analysis of dendritic branching pattern of granular cells from human dentate gyrus. Exp Neurol. 1976;52: 295–310. doi: 10.1016/0014-4886(76)90173-4 947769 10.1016/0014-4886(76)90173-4

[16] Lindsay RD, Scheibel AB. Quantitative analysis of the dendritic branching pattern of granule cells from adult rat dentate gyrus. Exp Neurol. 1981;73: 286–297. 7250282 10.1016/0014-4886(81)90062-5

[17] Jones TA, Schallert T. Overgrowth and pruning of dendrites in adult rats recovering from neocortical damage. Brain Res. 1992;581: 156–160. 1498666 10.1016/0006-8993(92)90356-e

[18] Lowndes M, Stewart MG. Dendritic spine density in the lobus parolfactorius of the domestic chick is increased 24 h after one-trial passive avoidance training. Brain Res. 1994;654: 129–136. doi: 10.1016/0006-8993(94)91578-4 7982084 10.1016/0006-8993(94)91578-4

[19] Biernaskie J, Corbett D. Enriched rehabilitative training promotes improved forelimb motor function and enhanced dendritic growth after focal ischemic injury. J Neurosci. 2001;21: 5272–5280. 11438602 10.1523/JNEUROSCI.21-14-05272.2001 PMC6762844

[20] Mouchet P, Yelnik J. Basic electrotonic properties of primate pallidal neurons as inferred from a detailed analysis of their morphology: A modeling study. Synapse. 2004;54: 11–23. doi: 10.1002/syn.20060 15300880 10.1002/syn.20060

[21] Uylings HBM, Van Pelt J, Verwer RWH. Topological analysis of individual neurons In: Capowski JJ, editor. Computer Techniques in Neuroanatomy. New York: Plenum; 1989 pp. 215–239.

[22] Horton RE. Erosional development of streams and their drainage basins; Hydrophysical approach to quantitative morphology. Bull Geol Soc Am. 1945;56: 275–370. doi: 10.1130/0016-7606(1945)56[275:EDOSAT]2.0.CO;2

[23] Strahler AN. Quantitative analysis of watershed geomorphology. Trans Am Geophys Union. 1957;38: 913–920.

[24] Horsfield K, Cumming G. Morphology of the bronchial tree in the dog. Respir Physiol. 1976;26: 173–182. 935696 10.1016/0034-5687(76)90095-5

[25] Horsfield K. Morphometry of the small pulmonary arteries in man. Circ Res. 1978;42: 593–597. doi: 10.1161/01.RES.42.5.593 639181 10.1161/01.res.42.5.593

[26] Jiang ZL, Kassab GS, Fung YC. Diameter-defined Strahler system and connectivity matrix of the pulmonary arterial tree. J Appl Physiol. 1994;76: 882–892. 8175603 10.1152/jappl.1994.76.2.882

[27] Tawhai MH, Hunter P, Tschirren J, Reinhardt J, McLennan G, Hoffman EA. CT-based geometry analysis and finite element models of the human and ovine bronchial tree. J Appl Physiol. 2004;97: 2310–2321. doi: 10.1152/japplphysiol.00520.2004 15322064 10.1152/japplphysiol.00520.2004

[28] Holland PG. The maintenance of structure and shape in three mallee Eucalypts. New Phytol. 1969;68: 411–421. doi: 10.1111/j.1469-8137.1969.tb06453.x

[29] Barker SB, Cumming G, Horsfield K. Quantitative morphometry of the branching structure of trees. J Theor Biol. 1973;40: 33–43. doi: 10.1016/0022-5193(73)90163-X 4723553 10.1016/0022-5193(73)90163-x

[30] McMahon TA, Kronauer RE. Tree structures: Deducing the principle of mechanical design. J Theor Biol. 1976;59: 443–466. doi: 10.1016/0022-5193(76)90182-X 957700 10.1016/0022-5193(76)90182-x

[31] Moon JW. On Horton’s Law for random channel networks. Ann Discret Math. 1980;8: 117–121. doi: 10.1016/S0167-5060(08)70860-4

[32] Yekutieli I, Mandelbrot BB. Horton-Strahler ordering of random binary trees. J Phys A Math Gen. 1994;27: 285–293. doi: 10.1088/0305-4470/27/2/014

[33] Yamamoto K, Yamazaki Y. Topological self-similarity on the random binary-tree model. J Stat Phys. 2010;139: 62–71. doi: 10.1007/s10955-010-9928-5

[34] Kirchner JW. Statistical inevitability of Horton’s laws and the apparent randomness of stream channel networks. Geology. 1993;21: 591–594.

[35] Hollingworth T, Berry M. Network analysis of dendritic fields of pyramidal cells in neocortex and Purkinje cells in the cerebellum of the rat. Philos Trans R Soc Lond B Biol Sci. 1975;270: 227–264. doi: 10.1098/rstb.1975.0008 239415 10.1098/rstb.1975.0008

[36] Binzegger T, Douglas RJ, Martin KAC. Axons in cat visual cortex are topologically self-similar. Cereb Cortex. 2005;15: 152–165. doi: 10.1093/cercor/bhh118 15238439 10.1093/cercor/bhh118

[37] Grueber WB, Jan LY, Jan YN. Tiling of the Drosophila epidermis by multidendritic sensory neurons. Development. 2002;129: 2867–2878. 12050135 10.1242/dev.129.12.2867

[38] Kim MD, Jan LY, Jan YN. The bHLH-PAS protein Spineless is necessary for the diversification of dendrite morphology of Drosophila dendritic arborization neurons. Genes Dev. 2006;20: 2806–2819. doi: 10.1101/gad.1459706 17015425 10.1101/gad.1459706 PMC1619948

[39] Jinushi-Nakao S, Arvind R, Amikura R, Kinameri E, Liu AW, Moore AW. Knot/Collier and Cut control different aspects of dendrite cytoskeleton and synergize to define final arbor shape. Neuron. 2007;56: 963–978. doi: 10.1016/j.neuron.2007.10.031 18093520 10.1016/j.neuron.2007.10.031

[40] Stewart A, Tsubouchi A, Rolls MM, Tracey WD, Sherwood NT. Katanin p60-like1 promotes microtubule growth and terminal dendrite stability in the larval class IV sensory neurons of Drosophila. J Neurosci. 2012;32: 11631–11642. doi: 10.1523/JNEUROSCI.0729-12.2012 22915107 10.1523/JNEUROSCI.0729-12.2012 PMC3495988

[41] Ferreira T, Ou Y, Li S, Giniger E, Van Meyel DJ. Dendrite architecture organized by transcriptional control of the F-actin nucleator Spire. Development. 2014;141: 650–660. doi: 10.1242/dev.099655 24449841 10.1242/dev.099655 PMC3899818

[42] Shimono K, Fujishima K, Nomura T, Ohashi M, Usui T, Kengaku M, et al An evolutionarily conserved protein CHORD regulates scaling of dendritic arbors with body size. Sci Rep. 2014;4: 4415 doi: 10.1038/srep04415 24643112 10.1038/srep04415 PMC3958717

[43] Van Pelt J, Van Ooyen A, Uylings HBM. Modeling dendritic geometry and the development of nerve connections In: De Schutter E, editor. Computational Neuroscience: Realistic Modeling for Experimentalists. Boca Raton, USA: CRC-Press; 2001 pp. 179–208.

[44] Watson HW, Galton F. On the probability of the extinction of families. J Anthropol Inst Gt Britain Irel. 1875;4: 138–144.

[45] Kliemann W. A stochastic dynamical model for the characterization of the geometrical structure of dendritic processes. Bull Math Biol. 1987;49: 135–152. doi: 10.1007/BF02459695 3607336 10.1007/BF02459695

[46] Cuntz H, Forstner F, Borst A, Häusser M. One rule to grow them all: A general theory of neuronal branching and its practical application. PLoS Comput Biol. 2010;6: e1000877 doi: 10.1371/journal.pcbi.1000877 20700495 10.1371/journal.pcbi.1000877 PMC2916857

[47] Cuntz H, Borst A, Segev I. Optimization principles of dendritic structure. Theor Biol Med Model. 2007;4: 21 doi: 10.1186/1742-4682-4-21 17559645 10.1186/1742-4682-4-21 PMC1924501

[48] Rall W. Electrophysiology of a dendritic neuron model. Biophys J. 1962;2: 145–167. doi: 10.1016/S0006-3495(62)86953-7 14490040 10.1016/s0006-3495(62)86953-7 PMC1366481

[49] Jaffe DB, Carnevale NT. Passive normalization of synaptic integration influenced by dendritic architecture. J Neurophysiol. 1999;82: 3268–3285. 10601459 10.1152/jn.1999.82.6.3268

[50] Van Pelt J, Schierwagen A. Electrotonic properties of passive dendritic trees—effect of dendritic topology In: Van Pelt J, Corner MA, Uylings HBM, Lopes da Silva FH, editors. Progress in Brain Research. Amsterdam: Elsevier; 1994 pp. 127–149. doi: 10.1016/S0079-6123(08)60536-1 7800809

[51] Shreve RL. Statistical law of stream numbers. J Geol. 1966;74: 17–37.

[52] Van Pelt J, Uylings HBM, Verwer RWH. Distributional properties of measures of tree topology. Acta Stereol. 1989;8: 465–470.

[53] Soni VH, Ketisch PM, Rodríguez JD, Shpunt A, Hübler AW. Topological similarities in electrical and hydrological drainage networks. J Appl Phys. 2011;109: 36103 doi: 10.1063/1.3533389

[54] Arenas A, Danon L, Díaz-Guilera A, Gleiser PM, Guimerà R. Community analysis in social networks. Eur Phys J B. 2004;38: 373–380. doi: 10.1140/epjb/e2004-00130-1

[55] Berry M, Bradley P. The growth of the dendritic trees of Purkinje cells in the cerebellum of the rat. Brain Res. 1976;112: 1–35. doi: 10.1016/0006-8993(76)90331-0 947479 10.1016/0006-8993(76)90331-0

[56] Yen L, Sibley JT, Constantine-Paton M. Analysis of synaptic distribution within single retinal axonal arbors after chronic NMDA treatment. J Neurosci. 1995;15: 4712–4725. 7540683 10.1523/JNEUROSCI.15-06-04712.1995 PMC6577734

[57] Liu QA, Shio H. Mitochondrial morphogenesis, dendrite development, and synapse formation in cerebellum require both Bcl-w and the glutamate receptor δ2. PLoS Genet. 2008;4: e1000097 doi: 10.1371/journal.pgen.1000097 18551174 10.1371/journal.pgen.1000097 PMC2405952

[58] Djagaeva I, Doronkin S. Dual regulation of dendritic morphogenesis in Drosophila by the COP9 signalosome. PLoS One. 2009;4: e7577 doi: 10.1371/journal.pone.0007577 19855832 10.1371/journal.pone.0007577 PMC2762029

[59] Djagaeva I, Doronkin S. COP9 limits dendritic branching via Cullin3-dependent degradation of the actin-crosslinking BTB-domain protein Kelch. PLoS One. 2009;4: e7598 doi: 10.1371/journal.pone.0007598 19859546 10.1371/journal.pone.0007598 PMC2762543

[60] Hakeda S, Suzuki T. Golden goal controls dendrite elongation and branching of multidendritic arborization neurons in Drosophila. Genes to Cells. 2013;18: 960–973. doi: 10.1111/gtc.12089 23919529 10.1111/gtc.12089

[61] Robain O, Wen GY, Wisniewski HM, Shek JW, Loo YH. Purkinje cell dendritic development in experimental phenylketonuria. Acta Neuropathol. 1981;53: 107–112. doi: 10.1007/BF00689990 7193957 10.1007/BF00689990

[62] Grueber WB, Graubard K, Truman JW. Tiling of the body wall by multidendritic sensory neurons in Manduca sexta. J Comp Neurol. 2001;440: 271–283. doi: 10.1002/cne.1385 11745623 10.1002/cne.1385

[63] Cuntz H, Forstner F, Borst A, Häusser M. The TREES toolbox—Probing the basis of axonal and dendritic branching. Neuroinformatics. 2011;9: 91–96. doi: 10.1007/s12021-010-9093-7 21222051 10.1007/s12021-010-9093-7 PMC7612393

[64] Werner C, Smart JS. Some new methods of topologic classification of channel networks. Geogr Anal. 1973;5: 271–295. doi: 10.1111/j.1538-4632.1973.tb00491.x

[65] Van Pelt J, Dityatev AE, Uylings HBM. Natural variability in the number of dendritic segments: Model-based inferences about branching during neurite outgrowth. J Comp Neurol. 1997;387: 325–340. 9335418

[66] Cuntz H, Forstner F, Haag J, Borst A. The morphological identity of insect dendrites. PLoS Comput Biol. 2008;4: e1000251 doi: 10.1371/journal.pcbi.1000251 19112481 10.1371/journal.pcbi.1000251 PMC2588660

[67] Cuntz H, Mathy A, Häusser M. A scaling law derived from optimal dendritic wiring. Proc Natl Acad Sci. 2012;109: 11014–11018. doi: 10.1073/pnas.1200430109 22715290 10.1073/pnas.1200430109 PMC3390826

[68] Beining M, Jungenitz T, Radic T, Deller T, Cuntz H, Jedlicka P, et al Adult-born dentate granule cells show a critical period of dendritic reorganization and are distinct from developmentally born cells. Brain Struct Funct. 2017;222: 1427–1446. doi: 10.1007/s00429-016-1285-y 27514866 10.1007/s00429-016-1285-y

[69] Budd JML, Kovács K, Ferecskó AS, Buzás P, Eysel UT, Kisvárday ZF. Neocortical axon arbors trade-off material and conduction delay conservation. PLoS Comput Biol. 2010;6: e1000711 doi: 10.1371/journal.pcbi.1000711 20300651 10.1371/journal.pcbi.1000711 PMC2837396

[70] Borst A, Haag J. The intrinsic electrophysiological characteristics of fly lobula plate tangential cells: I. Passive membrane properties. J Comput Neurosci. 1996;3: 313–336. doi: 10.1007/BF00161091 9001975 10.1007/BF00161091

[71] Barrett JN, Crill WE. Specific membrane properties of cat motoneurones. J Physiol. 1974;239: 301–324. 4137933 10.1113/jphysiol.1974.sp010570 PMC1330925

[72] Spruston N, Johnston D. Perforated patch-clamp analysis of the passive membrane properties of three classes of hippocampal neurons. J Neurophysiol. 1992;67: 508–529. 1578242 10.1152/jn.1992.67.3.508

[73] Roth A, Häusser M. Compartmental models of rat cerebellar Purkinje cells based on simultaneous somatic and dendritic patch-clamp recordings. J Physiol. 2001;535: 445–472. doi: 10.1111/j.1469-7793.2001.00445.x 11533136 10.1111/j.1469-7793.2001.00445.x PMC2278793

