# Supplementary figures and images for: Universal features of dendrites through centripetal branch ordering
Source: PLoS Comput Biol. 2017 Jul 3;13(7):e1005615. doi: 10.1371/journal.pcbi.1005615 (PMC5515450; doi:10.1371/journal.pcbi.1005615)

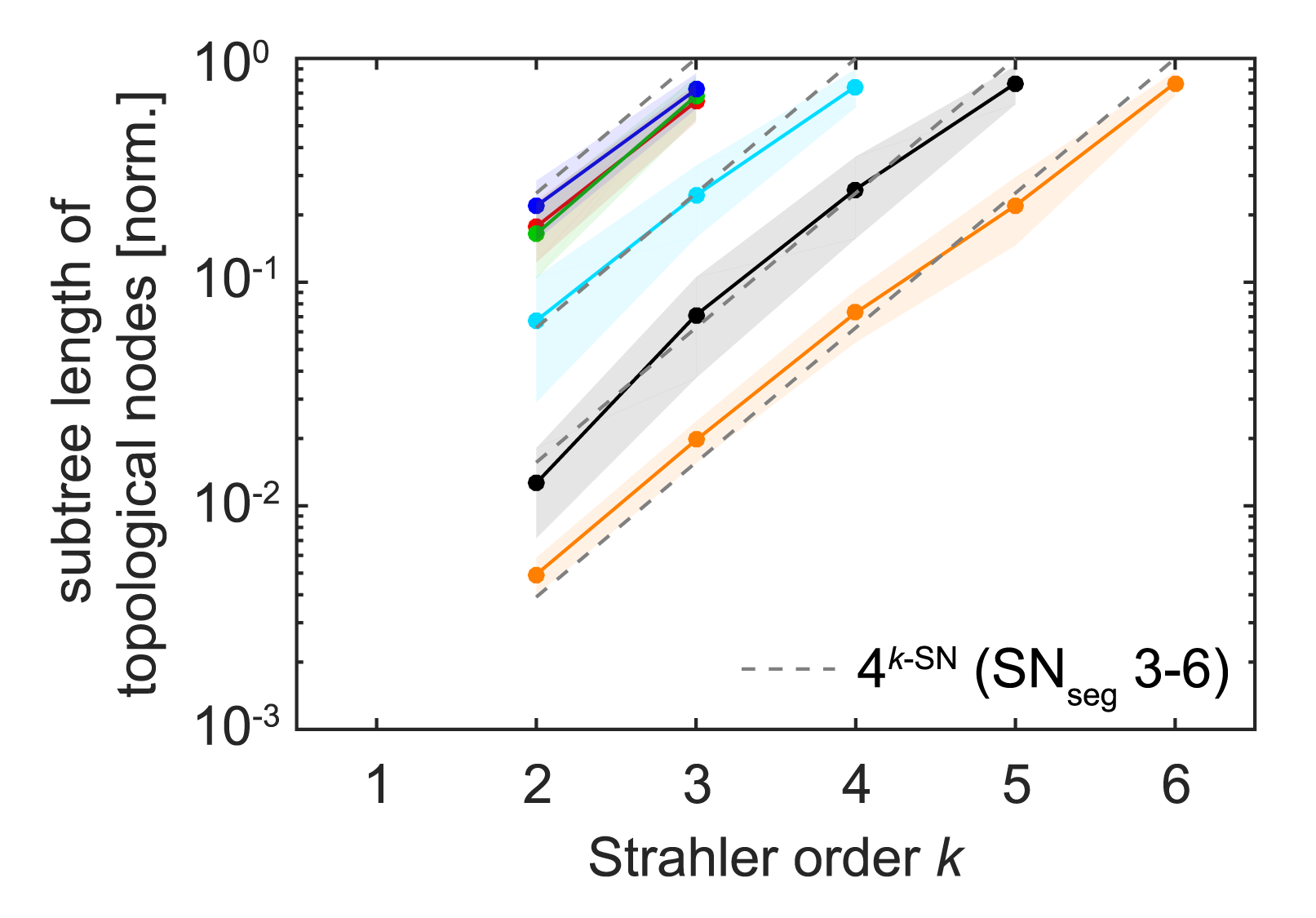

Supplement: S1 Fig — Average (lines with markers) and standard deviations (shaded areas) of normalised subtree lengths for all topological nodes as a function of SO in real dendritic trees. Colours indicate cell types (for legend see Fig 4A). Values were normalised by expressing them as a ratio of total dendritic length. Grey dashed lines same as in Fig 4E. (TIF) [file pcbi.1005615.s001.tif]
